# Fascia iliaca compartment block for analgesia in total hip replacement: A randomized controlled study protocol

**DOI:** 10.1097/MD.0000000000022158

**Published:** 2020-09-11

**Authors:** Jiannan Song, Yan Qiao, Qi Zhou, Xizhe Zhang

**Affiliations:** a Department of Anesthesiology; b Department of neurology, Chifeng Municipal Hospital, Inner Mongolia, China.

**Keywords:** analgesia, fascia iliaca compartment block, opioid, protocol, total hip replacement

## Abstract

**Background:**

Pain management after the total joint arthroplasty is still challenging, but worthy of attention, because good pain management can improve the outcomes of patient. It is still controversial whether fascia iliaca compartment block (FICB) can effectively decrease the opioid consumption and pain after total hip replacement (THR) owing to the number of published investigations is small. The purpose of this present study is to assess the efficacy and safety of FICB for postoperative analgesia after THR.

**Methods:**

This is a single center, placebo-controlled randomized trial which is performed in accordance with the SPIRIT Checklist for randomized studies. It was authorized via the Chifeng Municipal Hospital institutional review committee (H2020-19-8). 100 patients undergoing THR will be included in this study. Patients are randomly divided into 2 groups: FICB group or Non-FICB group, FICB with 5mgmL^–1^ of epinephrine and 40 mL of ropivacaine 0.2%. Primary outcomes are pain score at different time point. Visual analog scale is used to assess the pain (10: the maximum possible pain and 0: absent pain). The secondary outcomes are the postoperative complications, length of hospital stay and total consumption of opioid. All the needed analyses are implemented through utilizing SPSS for Windows Version 15.0.

**Results:**

Figure 1 will show the primary and secondary outcomes.

**Conclusion:**

This trial can provide an evidence for the use of FICB for analgesia after THR.

## 1 Introduction

Total hip replacements (THRs) are quite successful surgery for many end-stage hip diseases in terms of pain relief and functional recovery.^[1,2]^ And the demand for primary THR is projected to grow by 3.48 million procedures in the United States alone by 2030.^[3,4]^ However, THR are associated with severe perioperative pain which prolongs length of hospitalization and increases the morbidity and medical cost.^[5,6]^ Adequate pain management is crucial for improving the satisfaction of patients and clinical outcomes. Numerous strategies have been implemented to reduce postoperative pain following THR including local infiltration analgesia, epidural analgesia, intravenous non-steroidal anti-inflammatory agents and peripheral nerve block.^[7–9]^ Each method has its limitations and opioids are used as an alternative method to reduce pain. However, opioid can cause many side effects, such as gastrointestinal reactions, respiratory depression, retention of urine and constipation.^[10,11]^ Therefore, multimodal analgesia has been extensively used in perioperative period of THR.

Fascia iliaca compartment block (FICB) is a kind of anterior method to lumbar plexus. The hip joint is supplied via the branches of sciatic, obturator, and femoral nerves.^[12]^ They are all the part of lumbar plexus, which means that blocking lumbar plexus provides an elegant method for the postoperative analgesia after THR. FICB has no effect on muscle strength of quadriceps femoris and theoretically, it will not increase the risk of fall during rehabilitation. Several articles have suggested that FICB was related to the reduced consumption of opioid analgesics and the improvement of pain relief in patients with THR.^[13,14]^ However, others reported that there was no positive effects for postoperative pain management.

It is not clear that whether FICB is a kind of effective approach for the postoperative analgesia after THR. Therefore, we conduct this randomized controlled study protocol in order to evaluate the efficacy and safety of FICB for analgesia after THR. Our assumption is that FICB could reduce the consumption of opioid and pain score after operation in the early postoperative period.

## 2 Methods

This is a single center, placebo-controlled randomized trial which is performed in accordance with the SPIRIT Checklist for randomized studies. It was authorized via institutional review committee in the Chifeng Municipal Hospital (H2020–19-8) and then was registered in research registry (researchregistry5894). The orthopedic surgeon will explain the details of trial, afterward, patiently answer all the questions from patients. These patients are then presented with the written information of our trial. Each of patient received a written informed consent. Since all patients participated voluntarily, they could withdraw at any time during the trial.

### 2.1 Subjects

100 patients undergoing THR will be included in this study. In the random envelope, all participants will be assigned a random number via utilizing the random number table, and the result of allocation is hidden. Patients are randomly divided into 2 groups: FICB group (n = 50) or Non-FICB group (n = 50), FICB with 5mgmL^−1^ of epinephrine and 40 mL of ropivacaine 0.2%. Physicians, statisticians, data collectors and evaluators are all blinded to the allocation.

### 2.2 Inclusion and exclusion criteria

The inclusion criteria included patients aged between 55 and 70 years old, with ASA physical condition I-III, and receiving the total hip arthroplasty in our hospital. The exclusion criteria included refusal or inability to sign the informed consent, intolerance to any drug utilized in our study, BMI above 35 kg/m^2^, with the history of renal and hepatic dysfunction, with the clinical evidence of peripheral neuropathies, coagulation disorders, and opioid dependence.

### 2.3 Anesthesia

Patients are given intravenously 0.02mgkg^−1^ of midazolam and 0.5mgkg-^1^ of fentanyl. All patients will undergo the spinal anesthesia in lateral position. The level and midline of the intervertebral spaces of L3–4 and L4–5 are determined, afterward, spinal anesthesia is performed by injecting 10 mg 0.5% bupivacaine hyperbaric solution with the 25-gauge Quincke needle. Patients are placed promptly in supine position. After the addition of 18 intravenous cannula to the ankle, all the patients receive THR in an independent lateral position via a hip surgeon with fifteen years of experience. The patient is then transferred into a separate ward. After the standard monitoring, the patients in group FICB are performed with ultrasound; Afterward, both 2 groups of patients start the patient-controlled intravenous analgesia.

### 2.4 Block techniques

In supine position, the chlorhexidine and betadine are utilized to disinfect inguinal crease area. A 5 MHz-12 MHz linear probe is placed parallel to inguinal ligament at inguinal crease, and the fascia iliaca, fascia lata, femoral artery, femoral nerve as well as iliacus muscle are found. After the probe is rotated 90 to 135 degrees counterclockwise, we makes this probe parallel to vertebral axis. From this view, a 22G Tuohy needle, connected to a venous extension tube between syringe and the needle, is inserted in the plane and then pushed toward the iliacusmuscle and fascia iliaca. After we confirm that the needle passed through the fascia iliaca, we inject the local anesthetic prepared in advance. After the injection of normal saline, we utilize the hydrodissection technique under the guidance of real-time ultrasound. In hydrodissection technique, a small amount of the local anesthetic (1–2 mL) is injected, and then the needle is extended to proximal side for proximal local anesthetic diffusion. Through this hydrodissection process, the needle passes through the proximal end, goes deep into the fascia iliaca, and enters iliac fossa, and only enters space created via the fluid collection. Prior to the entire needle is inserted into skin, this hydrodissection technique need to repeat. And the total volume is 5mgmL^−1^ 0.2% epinephrine and 40 mL of ropivacaine.

### 2.5 Outcomes

Primary outcomes are pain score at different time point. Visual analog scale is used to assess the pain (10: the maximum possible pain and 0: absent pain).^[15]^ The secondary outcomes are the postoperative complications, length of hospital stay and total consumption of opioid.

### 2.6 Statistical analysis

All the needed analyses are implemented through utilizing SPSS for Windows Version 15.0. All the data are represented with proper characteristics as median, mean, percentage as well as standard deviation. Independent samples *t-*test or the Mann-Whitney *U* test is utilized for the comparison between groups. Chi-square detection is utilized to compare the categorical variables among the groups. A *P* < .05 is regarded the significant in statistics.

## 3 Results

**Figure 1:**
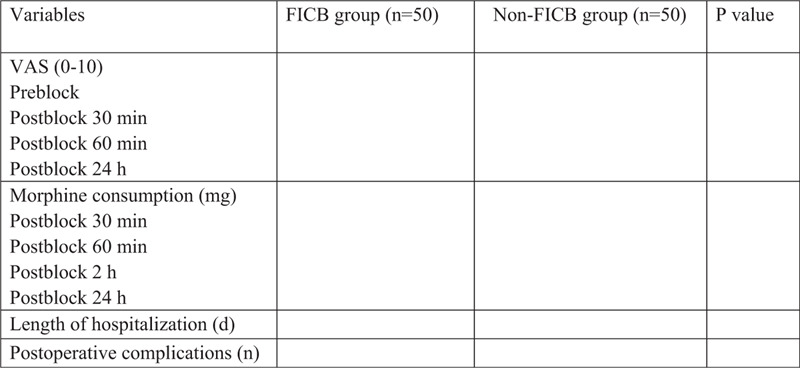
Primary and secondary outcomes between FICB group and non-FICB group. FICB = fascia iliaca compartment block.

Figure 1 will show the primary and secondary outcomes.

## 4 Discussion

Pain management after the total joint arthroplasty is still challenging, but worthy of attention, because good pain management can improve the outcomes of patient.^[16]^ About 6 to 10 percent of the patients may experience moderate to severe pain lasting at least 3 months after the operation, which is considered as the chronic postoperative pain.^[17]^ Different institutions have different postoperative treatment protocols, which may lead to different techniques used and patterns, and affecting long-term results and success. The lack of a definitive “gold standard” and various postoperative pain treat programs indicate that there is considerable room for improving the standard of care. The techniques of regional anesthesia can decrease postoperative pain scores and the consumption of opioid, so as to improve patient satisfaction and better treatment outcomes.^[18,19]^ It is still controversial whether FICB can effectively decrease the opioid consumption and pain after THR owing to the number of published investigations is small. We conduct this protocol to evaluate the safety and efficacy of FICB in postoperative analgesia after THR.

## 5 Conclusion

This trial can provide an evidence for the use of FICB for analgesia after THR.

## Author contributions

Xizhe Zhang design the protocol. Qi Zhou review and edit the study protocol. Yan Qiao perform conduction of experiments. Jiannan Song finish the manuscript.

**Investigation:** Yan Qiao.

**Methodology:** Yan Qiao.

**Writing – original draft:** Jiannan Song.

**Writing – review & editing:** Qi Zhou.
